# Synergistic Effect of Nickel and Cobalt Nanoparticles Anchored on Boron-Doped Reduced Graphene Oxide for Enhanced Alkaline Hydrogen Evolution Reaction

**DOI:** 10.1021/acsomega.5c08749

**Published:** 2025-11-20

**Authors:** Nida Arasan, Fatih Akkurt

**Affiliations:** Department of Chemical Engineering, Faculty of Engineering, 37511 Gazi University, Ankara 06570, Turkey

## Abstract

In this study, novel electrocatalysts based on boron-doped reduced graphene oxide (B-rGO-H) decorated with nickel (Ni) and cobalt (Co) nanoparticles were synthesized via a modified polyol method and evaluated for their hydrogen evolution reaction (HER) performance in alkaline media. The structural, morphological, and compositional properties of the synthesized materials swere thoroughly characterized using FTIR, Raman spectroscopy, XRD, XPS, SEM-EDS, and HR-TEM analyses. Electrochemical evaluations were carried out through linear sweep voltammetry (LSV), Tafel slope analysis, electrochemical impedance spectroscopy (EIS), and chronopotentiometry. Among the prepared catalysts, the NiCo@B-rGO-H hybrid demonstrated superior HER performance with a low overpotential of 242 mV at 10 mA cm^–2^, a favorable Tafel slope of 165 mV dec^–1^, and exhibits long-term electrochemical stability comparable to that of a 20% Pt@C commercial electrocatalyst over 24 h. The enhanced activity is attributed to the synergistic interaction between Ni and Co nanoparticles and the conductive, defect-rich B-doped graphene matrix, which collectively improve the electron transfer kinetics and increase the electrochemically active sites. These findings highlight the potential of NiCo@B-rGO-H as a promising, cost-effective, and durable noble-metal-free electrocatalyst for efficient hydrogen production via water splitting in alkaline conditions.

## Graphical abstract

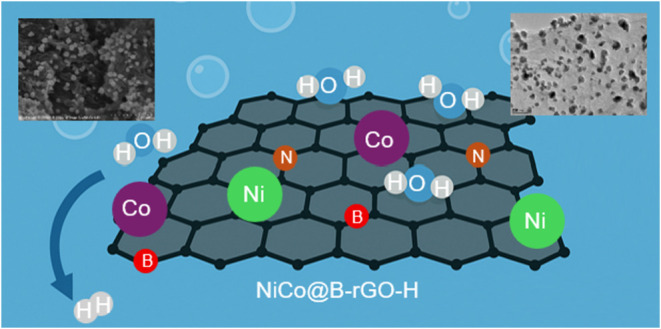

## 1 Introduction

Nowadays, energy demand has increased greatly as a result of the expanding global population and industrial growth. Ever-increasing fossil fuel use, with increasing energy demand, has brought air pollution and global warming issues caused by greenhouse gas emissions.^1^ Besides the concerns of global warming and climate change, there is an urgent need to develop clean and sustainable alternative energy sources to alleviate the effects of the upcoming energy crisis that we will face due to depleting fossil fuel reserves. Although some renewable energy sources, such as wind and solar energy, have been extensively researched, spatial and temporal problems are encountered regarding the storage and usage of these energy sources. In other words, renewable energy sources like solar and wind energy are not always available and everywhere. For this reason, new developments and approaches are being sought to transform the generated electrical energy obtained through renewable sources. Hydrogen stands out as a good alternative to conventional fossil fuels due to its high gravimetric energy density and environmentally friendly combustion products. Today, the majority of hydrogen is produced by reforming natural gas and gasifying coal, which contributes to greenhouse gas emissions. In this context, H_2_ production by electrochemical water splitting has emerged as a promising approach, considering the benefits of producing high-quality hydrogen while also being a clean and sustainable technique.^2,3^ Two half-reactions, the hydrogen evolution reaction (HER) and the oxygen evolution reaction (OER), are involved in the electrochemical splitting of water to produce hydrogen. OER happens at the anode while HER happens at the cathode of the electrolyzer. The anode and cathode electrocatalyst characteristics play an essential role in H_2_ production with high efficiency. Platinum (Pt) and iridium dioxide (IrO_2_) have shown high electrocatalytic activity for HER and OER up until now.^4^ However, their restricted availability and high cost have limited their large-scale production and application as electrocatalysts for electrochemical water splitting. For this reason, studies have been carried out on transition metals and their compounds, like transition metal oxides, chalcogenides, nitrides, phosphides, sulfides, and carbides, as an alternative to expensive noble metal-based electrocatalysts^4^ In addition to transition metal electrocatalysts, metal-free electrocatalysts, and hybrid electrocatalysts have been developed for HER.

Carbon-based materials such as graphene, carbon nanotubes, and carbon quantum dots have been extensively used for energy conversion and storage applications^5^ Recently, graphene, which is a 2D layered carbon-based material, has attracted significant interest due to its high electrical conductivity, high surface area, and tunable surface properties.^6^ The electrocatalytic performance of graphene can be improved by doping/codoping with nonmetal heteroatoms (N, S, P, B, etc.), defect engineering, and hybrid development.^7^ In this regard, doping heteroatoms into graphene is one of the most successful methods for developing the electronic characteristics of graphene by creating ’n’ or ’p’ type conductivity.^8^ Doping heteroatoms into graphene may produce defects due to the unequal charge distribution in neighboring regions. Such defects can efficiently enable and speed up charge transfer between neighboring carbon atoms, increasing the electrochemical performance of graphene materials doped with heteroatoms.^7^ In addition to improving electrical properties through heteroatom doping into graphene, new active sites can be introduced into the structure to adsorb atomic or molecular components that undergo electrochemical reactions.^7^ Sathe et al. have synthesized a B-substituted defective graphene electrocatalyst by using BH_3_-THF as a borylating agent with a facile wet chemical synthetic method and showed that B-doped graphene is an efficient metal-free electrocatalyst for HER with a lower overpotential compared to defective graphene. Raman spectroscopy and electrochemical impedance spectroscopy studies have revealed that boron doping has changed the electronic structure of graphene with more defect sites and lower charge transfer resistance.^9^ Li et al. have developed N and S codoped graphene electrocatalysts for HER and further activated the developed electrocatalyst with ZnCl_2_ to achieve a high specific surface area, high porosity, and defect-rich structure. It is reported that both codoping and chemical activation significantly improve the electrocatalytic performance of graphene toward HER.^10^ Coordination of transition metal atoms with carbon (C) and nonmetals can create highly active composites, which increase the HER activity of transitional metal-based catalysts. Zhang et al. have developed cobalt/Nitrogen-doped graphene catalysts to achieve high performance in the hydrogen evolution reaction. It is found that reaction kinetics were improved by the formation of Co–N and Co–C active sites on the defect-rich graphene layer.^11^ Alloying one transition metal with another can enhance the electrocatalytic activity due to a synergistic effect. Kamali et al. have developed a series of NiM/rGO alloys consisting of Ni and a nonprecious metal (M: Co, Fe, Mn, Cr, Cu, and Zn), and their activities have been tested for the hydrogen evolution reaction in alkaline media. The Ni,Co/rGO electrocatalyst showed the highest activity among the NiM/rGO series that had been developed. The overpotential for NiCo/rGO is significantly lower than that for the Ni/rGO electrocatalyst.^12^

In this study, we investigate the effect boron doping to the graphene structure and of adding Co nanoparticles on the electrochemical HER activity of boron-doped graphene-based Ni nanoparticles electrocatalyst in an alkaline solution. Boron doping provides a distinct electronic modulation compared to other heteroatoms such as nitrogen or sulfur. The incorporation of boron atoms into the graphene lattice introduces electron-deficient sites that can effectively tune the charge density of neighboring carbon atoms, facilitating proton adsorption and improving the overall kinetics of the hydrogen evolution reaction. This electron redistribution not only enhances the intrinsic activity of the Ni–Co active centers but also strengthens the metal–support coupling, stabilizing the catalytic interface under alkaline conditions. To the best of our knowledge, such a combination of boron-doped rGO support and bimetallic NiCo nanoparticles synthesized via a modified polyol method has not been previously reported, representing a novel approach toward designing conductive and defect-engineered hybrid electrocatalysts for alkaline HER. Boron-doped graphene-based Ni and Co nanoparticles containing electrocatalysts were prepared using the modified polyol method. An experimental system consisting of a standard three-electrode system connected to a potentiostat was used to test the synthesized electrocatalysts. The performances of the synthesized electrocatalysts were evaluated using linear sweep voltammetry, cyclic voltammetry, and electrochemical impedance spectroscopy techniques. FTIR, RAMAN, XRD, XPS, SEM-EDS, and HR-TEM analyses were performed within the scope of characterization studies.

## 2 Experimental Section

### 2.1 Chemicals

Graphite powder (99.0 wt %; Sigma-Aldrich), Sulfuric acid (95–98 wt %; Merck), Potassium permanganate (99.0 wt %; Merck), Sodium nitrate (99.0 wt %; Merck), Hydrogen peroxide (30 wt %; Tekkim), Hydrochloric acid (37 wt %; Merck), Barium chloride (99.0 wt %; Merck), Sodium borohydride (96.0 wt %; Merck), Sodium hydroxide (99.0 wt %; Merck), Boric acid (99.0 wt %; Merck), Ethylene glycol (99.8 wt %; Sigma-Aldrich), Hydrazine hydrate (80 wt %; Merck), Nickel­(II) nitrate hexahydrate (99.999 wt %; Sigma-Aldrich), Cobalt­(II) nitrate hexahydrate (99.0 wt %; Merck), Nafion dispersion (5 wt %; Sigma-Aldrich), Pt/C (20 wt %; ElectroChem), Potassium hydroxide (99.0 wt %; Merck), absolute ethanol (99.9 wt %; Merck) and deionized (DI) water were supplied. All chemical reagents were used as received without further purification.

### 2.2 Preparation of GO

GO was synthesized using the classical Hummers’ method.^13^ First, graphite powder (1 g) and NaNO_3_ were added to concentrated H_2_SO_4_ (25 mL) at an ice bath. Then KMnO_4_ (3 g) was slowly added into the mixture, it was mixed for 30 min in an ice bath and then at room temperature. Deionized water (45 mL) was added drop by drop into the mixture and stirred for another 30 min. After stirring, deionized water (150 mL) and H_2_O_2_ (30%; 8.5 mL) were added, and the procedure was completed. Finally, the product was filtered and washed several times with HCl (5%) and deionized water.

### 2.3 Preparation of rGO-H and B-rGO-H

The synthesis of boron-doped reduced graphene oxide was carried out using the hydrothermal method, which carried out the boron doping and reduction processes simultaneously. For hydrothermal reduction and boron doping, 400 mg rGO was added to 200 mL of deionized water and mixed for half an hour. Then, ultrasonic treatment was applied to the mixture for 1 h. Boric acid was used as a boron source. Boric acid was added to the GO solution and stirred for 30 min. The resulting mixture was placed in a 300 mL hydrothermal autoclave with a Teflon inner chamber, and hydrothermal treatment was applied in the oven at 180 °C for 12 h. The material obtained from the hydrothermal process was deionized, filtered, and washed several times with deionized water. The resulting product was named B-rGO-H. For comparison purposes, the same processes were repeated by hydrothermal reduction of graphene oxide without using a boron source, and the resulting material was named rGO-H.

### 2.4 Preparation of NiCo@B-rGO-H Electrocatalysts

Ni@B-rGO-H, Co@B-rGO-H, and NiCo@B-rGO-H electrocatalysts containing 15% metal at the beginning of the synthesis were synthesized using the modified polyol synthesis method. During the synthesis, ethylene glycol as a polyol, hydrazine as a reducing agent, nickel­(II) acetate tetrahydrate (Ni­(OCOCH_3_)_2_·4H_2_O), and cobalt­(II) acetate tetrahydrate (Co­(OCOCH_3_)­2·4H_2_O) metal salts as sources of nickel and cobalt have been used. For the modified polyol method, 100 mg of B-rGO is taken into a beaker, and 50 mL of ethylene glycol is added and mixed for half an hour. Simultaneously, a certain amount of metal salt is taken into a beaker, and 10 mL of ethylene glycol is added and mixed for half an hour. Under continuous stirring, the solution containing the metal salt is added dropwise to the B-rGO solution and stirred for 1 night. NaOH solution prepared in ethylene glycol is added dropwise to the mixture, and the pH value is adjusted to around 11–12. Five ml of hydrazine hydrate is added dropwise to the mixture and mixed for half an hour. The resulting mixture is placed in a steel autoclave with a Teflon inner chamber and kept at 200 °C for 6 h. The material obtained as a result of the synthesis is washed first with acetone and then with deionized water by centrifugation to remove impurities. Figure 1 shows the synthesis procedure for the NiCo@B-rGO-H electrocatalyst

**Figure 1:**
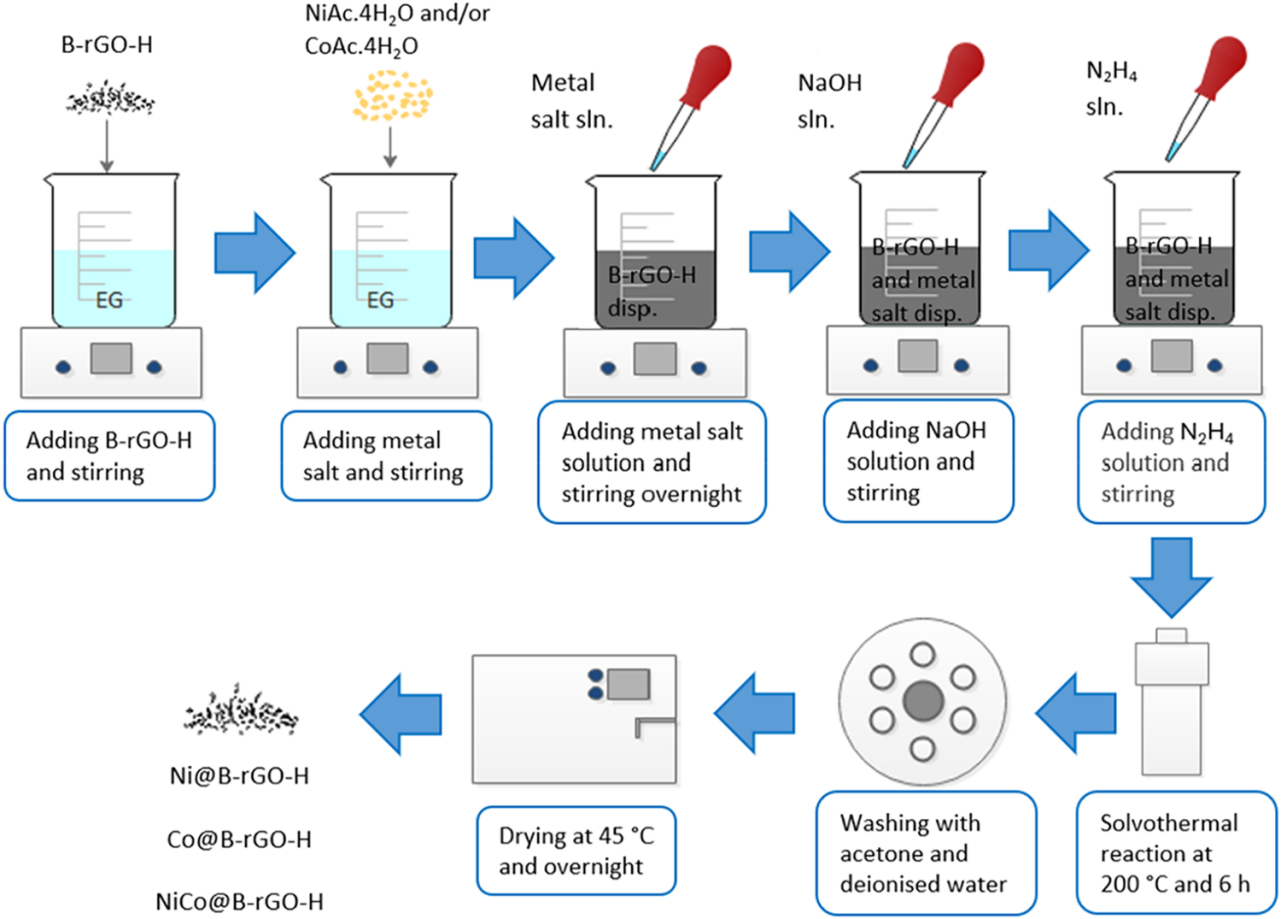
Synthesis procedure for NiCo@B-rGO-H electrocatalysts.

### 2.5 Characterization

The structural characterization of the as-synthesized products was carried out with FTIR, Raman, XRD, XPS, SEM, and TEM characterization techniques. Fourier Transform Infrared (FTIR) spectroscopy analysis performed in the wavenumber range of 500–4000 cm^–1^ was conducted using a Jasco FTIR spectrometer to identify the functional groups present in the synthesized electrocatalysts. To determine the structural properties of the materials in comparison to graphite, Raman spectroscopy analysis was carried out using a Renishaw inVIA system over the range of 500–3500 cm^–1^. The crystalline structure of the electrocatalysts was assessed using X-ray diffraction (XRD) spectroscopy, using a Rigaku Ultima-IV X-ray diffractometer with a Cu–Kα (λ_Cu–Kα_ = 0.154 nm) radiation in the 2θ range of 10–90°. To further support the results obtained from FTIR and XRD analyses, X-ray photoelectron spectroscopy (XPS) was performed using a PHI 5000 VersaProbe (K-Alpha) instrument to determine the valence states and surface composition of metals incorporated into the metal-containing electrocatalysts. The CasaXPS program was used to deconvolute the raw XPS data. To account for the charging effect, all spectra were corrected to the binding energy of C 1s at 285 eV. After carbon correction, all spectra were fitted using a nonlinear least-squares approach with a Shirley-type background and a mixed Gaussian–Lorentzian peak profile. Scanning electron microscopy (SEM) and transmission electron microscopy (TEM) images were obtained by QUANTA 400F Field Emission SEM instrument at 20 kV and Jem Jeol 2100F HRTEM instrument at 80 kV.

### 2.6 Electrochemical Measurements

All the electrochemical performance of as-prepared electrocatalyst was studied in alkaline water solution (1 M KOH) at an electrochemical workstation (Methrohm potentiostat/galvanostat) with three electrodes was set up with a Glassy Carbon (3 mm in diameter) electrode as the working electrode, Hg/HgO (1 M KOH) as the reference electrode, and graphite rod (3 mm in diameter) as a counter electrode. The as-prepared electrocatalysts were coated on a GC working electrode by the dropping method. For a typical coating procedure, 5 mg as-prepared electrocatalyst powder was dispersed in a solution containing 250 μL of ethanol, 700 μL of deionized water, and 50 μL of Nafion solution (5 wt %). The prepared catalyst ink was homogenized using ultrasonication and vortexing. Subsequently, 3 μL of homogenized catalyst ink was coated by dropping it onto the cleaned GCE surface and allowed to dry at room temperature. Electrochemical measurements were performed in 1 M KOH solution by linear sweep voltammetry (LSV), electrochemical impedance spectroscopy (EIS), cyclic voltammetry, and chronopotentiometry techniques. During the linear sweep voltammetry (LSV) measurements, all experiments were conducted at a controlled temperature of 25 °C with a stirring rate of 50 rpm to ensure homogeneous mass transfer in the electrolyte. Electrochemical impedance spectroscopy (EIS) measurements were performed under the same temperature conditions (25 °C) but without stirring, in order to avoid any hydrodynamic influence on the impedance response. In addition, all measurements were carried out in triplicate to ensure the reliability and reproducibility of the obtained data. LSV measurements were carried out at a scan rate of 5 mV/s. All potentials reported in this study were calibrated against the reversible hydrogen electrode (RHE). Accordingly, in alkaline media, the relationship is given by *E*_RHE_ = *E*_Hg/HgO_ + 0.924 V. The overpotential was defined as the potential at which the current density reached 10 mA cm^–2^. The Tafel equation (η = *a* + *b* log *j*) was used to determine the Tafel slopes from LSV data, where η is the overpotential, *a* is the empirical coefficient, *b* is the Tafel slope, and *j* is the current density, respectively. Tafel slope is closely related to the HER mechanism. In alkaline media, the hydrogen evolution reaction (HER) typically proceeds through a multistep process involving the Volmer, Heyrovsky, and Tafel reactions.^14^ The initial step, known as the Volmer reaction, corresponds to the electrochemical discharge of water molecules on the electrode surface. This is followed either by the Heyrovsky reaction, representing the electrochemical desorption of hydrogen, or by the Tafel reaction, where two adsorbed hydrogen atoms combine to form molecular hydrogen. The Volmer, Heyrovsky, and Tafel reaction steps for the hydrogen evolution reaction (HER) in alkaline medium are given in eqs 1–3 where M and MH_ads_ represent the electrode surface and the hydrogen adsorbed on the electrode surface, respectively.^15^

1 $$\mathrm{Volmer}:H_{2}O+M+e^{-}\to {\mathrm{MH}}_{\mathrm{ads}}+{\mathrm{OH}}^{-}$$

2 $$\mathrm{Heyrovsky}:H_{2}O+{\mathrm{MH}}_{\mathrm{ads}}+e^{-}\to H_{2}+M+{\mathrm{OH}}^{-}$$

3 $$\mathrm{Tafel}:2{\mathrm{MH}}_{\mathrm{ads}}\to 2M+H_{2}$$

EIS measurements were carried out in the frequency range of 100 kHz to 0.1 Hz. Experimental EIS data were fitted with the Nova software, and equivalent circuits were obtained. The stability of NiCo@B-rGO-H was evaluated through continuous CV cycling for 1000 cycles between −1.1 and −1.3*V*_Hg/HgO_ at 50 mV/s, complemented by chronopotentiometry tests. The LSV curves were obtained before and after 1000 CV cycles. The chronopotentiometry test of NiCo@B-rGO-H was conducted at a constant current density of 10 mA cm^–2^ (η_10_) for 24 h.

## 3 Results and Discussion

### 3.1 Structure and Morphology

Figure 2 presents the FT-IR spectra for graphite, graphene oxide (GO), and reduced graphene oxides (rGO-H and B-rGO-H). By interpreting the FT-IR spectrum of GO, the oxygen-containing functional groups attached to its surface were identified. Carboxyl groups are generally located at the edges in a typical GO structure, while hydroxyl and epoxy groups are found within the basal plane. The broad band at 3425 cm^–1^ corresponds to −OH stretching.^10^ The bands at 2924 and 2862 cm^–1^ are attributed to C–H stretching.^16^ The peak at 1720 cm^–1^ is associated with C=O stretching in carbonyl groups.^17^ The peak at 1620 cm^–1^ corresponds to C=C bonds, while the band at 1049 cm^–1^ is related to C–O stretching in carboxyl and epoxy groups.^17^ When comparing the FT-IR spectrum of graphite with that of GO, the presence of oxygen-containing functional groups in GO supports the successful synthesis of GO via Hummer’s method. Upon examining the FTIR spectra of rGO-H and B-rGO-H, it is evident that the C–O and C–O–C functional groups have disappeared from the structure, while the intensities of the −OH, C–OH, and C=O bands have significantly decreased. This observation supports the conclusion that GO has been successfully reduced. The B–C functional group is typically observed in the range of 1050 to 1200 cm^–1^.^18^ In the FTIR spectrum of B-rGO, a new band around 1111 cm^–1^ has been attributed to B–C stretching, indicating that boron atoms have been successfully doped into the carbon structure. The low intensity of the observed peak suggests that the level of boron doping is relatively low.

**Figure 2:**
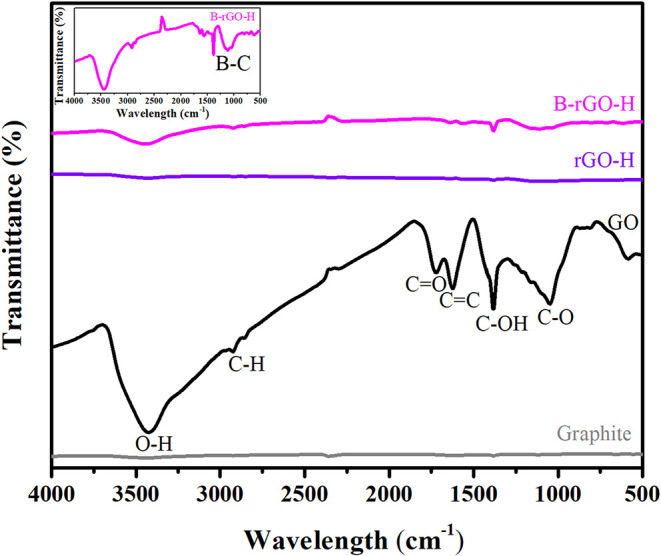
FTIR spectra of graphite, GO, rGO-H, and B-rGO-H materials.

Raman measurements have been conducted to describe the structural changes associated with the formation and reduction of graphene oxide (GO). The Raman spectra for GO, reduced graphene oxide (rGO-H), and boron-doped reduced graphene oxide (B-rGO-H) were compared with those of raw graphite, as illustrated in Figure 3. In carbon-based materials, the D, G, and 2D bands are particularly significant for determining the structural properties of graphite and its derivatives. The D band signals disorder among carbon atoms (indicating defects or oxygen-containing functional groups). Its presence suggests transitions between sp^2^ (ordered graphitic structure) and sp^3^ (disordered structure) hybridizations.^19^ The intensity of the D band correlates with the density of disorder or defects in the material; thus, for graphite and high-quality graphene, the D band intensity is typically low. The G band corresponds to symmetric vibrations between sp^2^ hybridized carbon atoms and reflects the ordered structure of graphite.^19^ Its intensity is related to the regularity of carbon–carbon bonds. The 2D band represents the multilayer structure of graphene and interactions between layers; its shape and width provide insights into the ordering of graphene layers.^20^ The ratio of *I*_D_/*I*_G_ serves as a measure of disorder within the structure, while the ratio of *I*_2D_/*I*_G_ indicates the number of layers in graphene-derived materials. A high intensity of the 2D band is characteristic of monolayer graphene.^21^ In raw graphite’s Raman spectrum, strong peaks at 1573 cm^–1^ (G band) and 2700 cm^–1^ (2D band) are observed, with a low-intensity peak at 1338 cm^–1^ for the D band, confirming its highly ordered structure (*I*_D_/*I*_G_ = 0.08). In contrast, GO displays peaks at 1597 cm^–1^ and 1348 cm^–1^ corresponding to the G and D bands, respectively (*I*_D_/*I*_G_ = 0.86). Compared to graphite, GO shows increased intensity in the D band, indicating higher disorder due to oxidation.^22^ The shift of the G band to a higher frequency supports the incorporation of oxygen functional groups into its structure. The increase in *I*_D_/*I*_G_ ratio further confirms that oxidation has introduced more disorder through oxygen functionalities. For rGO-H (*I*_D_/*I*_G_ = 0.96) and B-rGO-H (*I*_D_/*I*_G_ = 1.06) samples, the D and G bands appear at wavenumbers around 1345 and 1591 cm^–1^ for rGO-H and 1343 and 1589 cm^–1^ for B-rGO-H, respectively. These shifts toward lower frequencies compared to GO suggest that reduction processes have effectively removed oxygen functional groups from the structure.^23^ When comparing these Raman spectra with that of graphite, it is evident that GO, rGO-H, and B-rGO-H exhibit broader D and G bands, indicating lower crystallinity, consistent with X-ray diffraction (XRD) results. Notably, when comparing rGO-H and B-rGO-H spectra, an increase in *I*_D_/*I*_G_ from 0.96 to 1.06 suggests that boron incorporation may disrupt symmetry further, leading to increased disorder.^24^ The peak corresponding to the 2D band at 2700 cm^–1^ in graphite indicates its stacked and ordered structure; however, in GO, rGO-H, and B-rGO-H spectra, an expanded and distorted 2D band signifies defect presence. The number of graphene layers can be inferred from the ratio of *I*_2D_/*I*_G_. For monolayer graphene, this ratio is typically between 2 and 3; for bilayer graphene, it ranges from 1 to 2; while for multilayer graphene, it falls below 1.^24^ In this study, *I*_2D_/*I*_G_ ratios less than 1 were observed for GO, rGO-H, and B-rGO-H samples, indicating their multilayer graphene structures. This finding is corroborated by XRD analyses. A summary of Raman analysis results is presented in Table 1.

**Figure 3:**
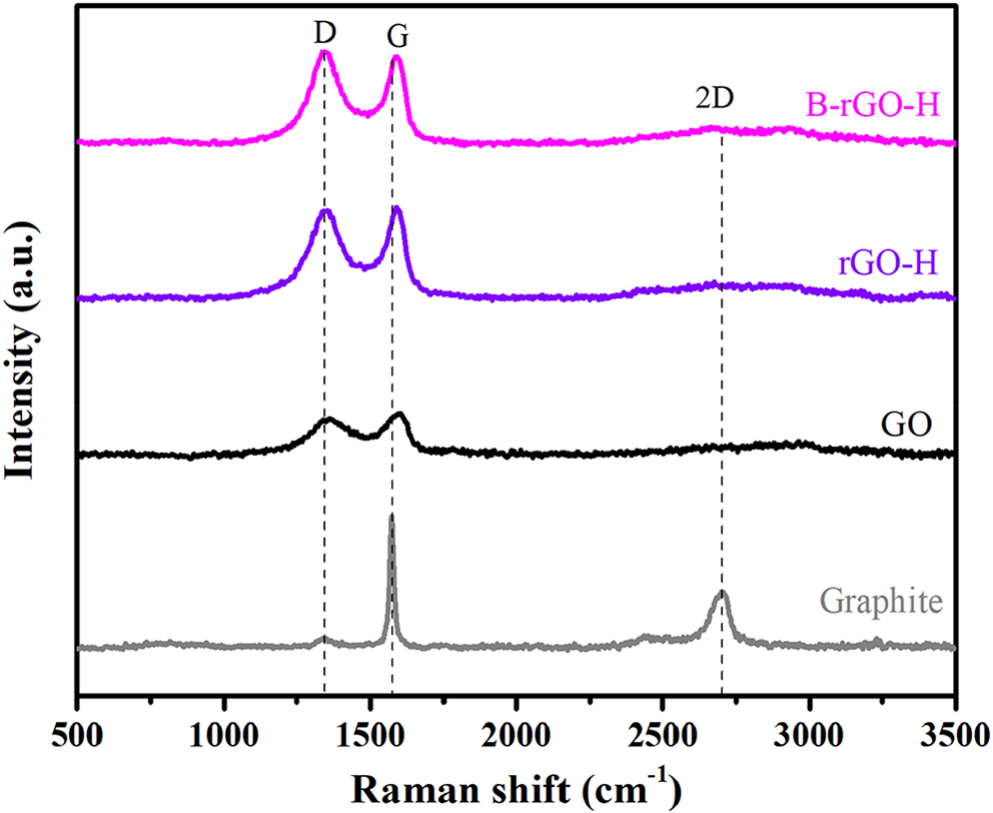
Raman spectra of graphite, GO, rGO-H, and B-rGO-H.

**Table 1:** Raman Analysis Summary of Graphite, GO, rGO-H, and B-rGO-H

		Raman shift (cm^–1^)				
Nu.	material	*D*	*G*	*2D*	*I*_D_/*I*_G_	*I*_2D_/*I*_G_
1	graphite	1338	1573	2700	0.08	2.02
2	GO	1348	1597		0.89	<1
3	rGO-H	1345	1591		0.96	<1
4	B-rGO-H	1343	1589		1.06	<1

X-ray diffraction analysis was performed to determine the crystal phases present in the synthesized materials. Figure 4 displays the XRD patterns for graphite, graphene, GO, rGO-H, and B-rGO-H. Crystal size (Lc) and interlayer spacing (d) parameters, which were determined using Scherrer’s Equation and Bragg’s Law, are presented in Table 2. In the XRD pattern of graphite, a strong, sharp peak is observed at 2θ = 26.5°, along with a weaker peak at 2θ = 54.6°, which correspond to the (002) and (004) planes of graphite, respectively.^25^ The interlayer spacing for graphite was determined to be 3.4 Å, and the crystallite size was calculated to be 217 Å. In the XRD pattern of graphene oxide (GO), two peaks are observed at 2θ = 12.0° and 2θ = 42.4°. The interlayer spacing for graphite is determined to be 7.3 Å, and the crystallite size is calculated to be 103.5 Å. The increase in interlayer spacing of GO compared to graphite supports the successful incorporation of oxygen-containing functional groups into the structure. In the literature, the broad diffraction peak typically located around 25° and the diffraction peak around 44° for reduced graphene oxide (rGO-H) have been associated with reflections from the (002) and (100) planes of graphene, respectively.^26^ For the synthesized rGO-H material, diffraction patterns were observed at angles of 2θ: 24.20°; 43.7°, respectively. The XRD analysis of B-rGO-H revealed characteristic diffraction patterns at angles of 2θ: 25.1°; 43.7°. The observed diffraction patterns are consistent with the characteristic peaks of rGO and indicate that GO was simultaneously reduced to rGO through boron doping as a result of the applied hydrothermal process.

**Figure 4:**
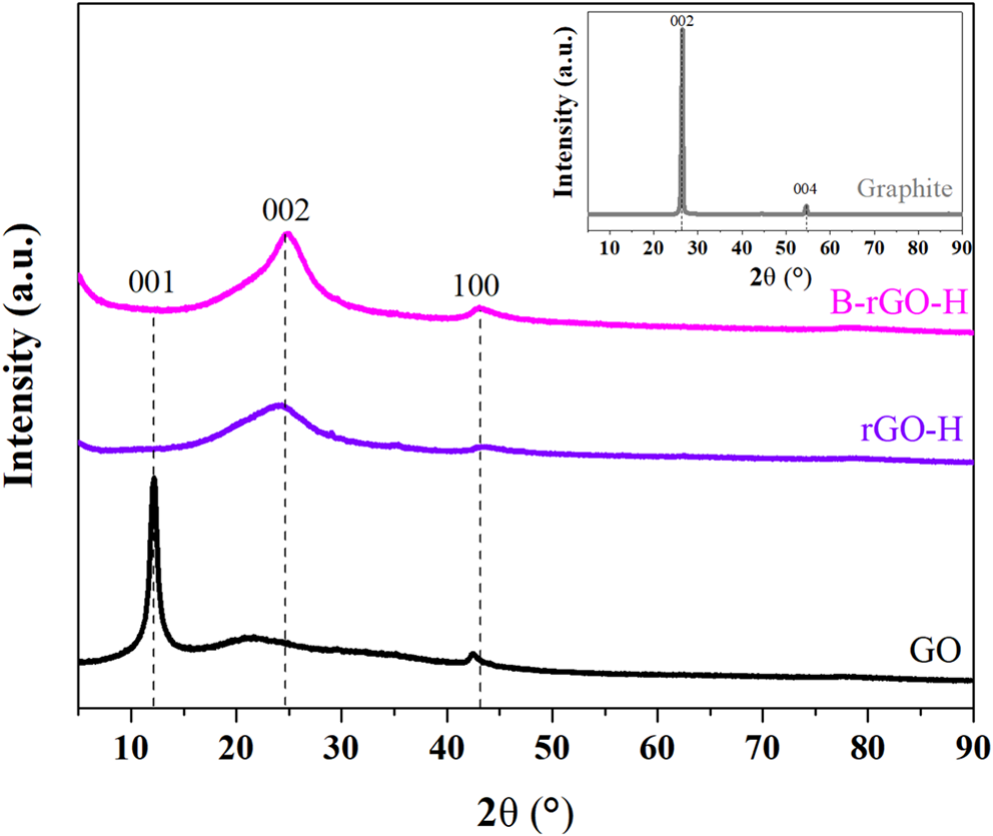
Wide-angle XRD spectra of Graphite, GO, rGO-H, and B-rGO-H

**Table 2:** XRD Analysis Summary of Graphite, rGO-H, and B-rGO-H

Nu.	material	cyristal size, *L*_c_ (Å)	interlayer spacing, *d* (Å)
1	graphite	217	3.4
2	GO	103.5	7.3
4	rGO-H	11.8	3.7
5	B-rGO-H	12.8	3.5

The objective of the synthesized B-rGO-H supported electrocatalysts is to incorporate nickel (Ni) and cobalt (Co) metals in the form of metallic nanoparticles. In the literature, the XRD pattern for the face-centered cubic phase of nickel (Ni) shows characteristic peaks at 44.66, 52.07, 76.65, 92.80, and 98.75° (JCPDS: 4-0850), which are associated with reflections from the (111), (200), (220), (311), and (222) planes.^27,28^ Similarly, the XRD pattern for the face-centered cubic phase of cobalt (Co) exhibits characteristic peaks at 44.22, 51.52, and 75.85° (JCPDS: 15-0806), corresponding to reflections from the (111), (200), and (220) planes.^29–31^ Figure 5 presents the wide-angle XRD spectra for the B-rGO-H supported electrocatalysts containing Ni and/or Co. As observed, no distinct characteristic peaks corresponding to metallic Ni or Co phases are evident in the XRD spectra of Ni@B-rGO-H and Co@B-rGO-H-5, electrocatalysts. Likewise, no significant characteristic peaks related to the oxide or hydroxide phases of Ni and Co metals were detected. The absence of distinct characteristic peaks for Ni and Co metals in these electrocatalysts may be attributed to their amorphous structure, small particle sizes, or low incorporation levels into the structure. Therefore, XPS analysis was conducted in addition to XRD analysis to gain insights into whether the metals were incorporated into the structure.

**Figure 5:**
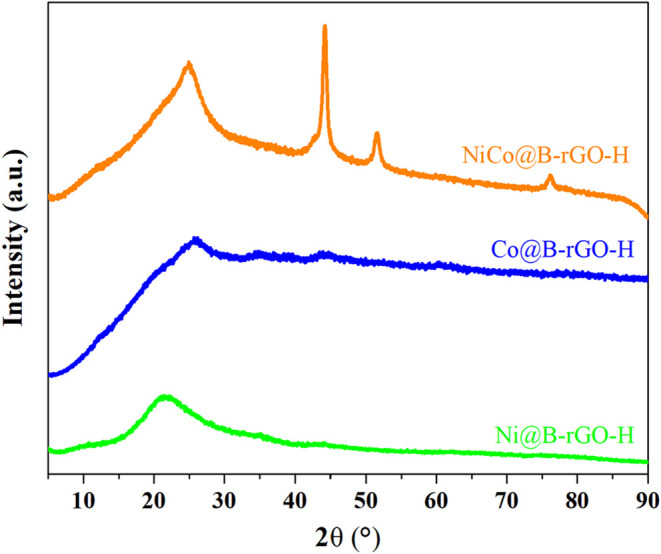
Wide-angle XRD spectra of Ni@B-rGO-H, Co@B-rGO-H, and NiCo@B-rGO-H electrocatalysts.

Figure 6 presents the wide-angle XRD spectra for the B-rGO-H and NiCo@B-rGO-H electrocatalysts for comparison. In the XRD analysis of the NiCo@B-rGO-H electrocatalyst, characteristic diffraction patterns were observed at around 2θ: 44.5, 52.4, and 76.1°. These diffraction patterns are consistent with the characteristic peaks of cubic Ni and Co phases. Due to the similar peak positions of cubic Ni and Co phases, it is challenging to distinguish between the peaks; therefore, the observed peaks were correlated with the (111), (200), and (220) planes of cubic Ni and Co based on literature references.^32–35^ The presence of Ni and Co in the electrocatalyst structure was subsequently confirmed through XPS analysis. No characteristic peaks corresponding to the oxide phases of Ni and Co, such as NiO, Ni_2_O_3_, Ni_3_O_4_, CoO, Co_2_O_3_, Co_3_O_4_, Ni­(OH)_2_, and Co­(OH)_2_, were observed in the XRD spectra of the Ni, Co@B-rGO-H electrocatalyst. This finding indicates that the majority of Ni and Co present in these electrocatalysts is in a metallic form. In contrast, no distinct characteristic peaks for metallic Ni and Co were observed in the XRD spectra of B-rGO-H supported electrocatalysts containing only Ni or only Co. The observation of characteristic peaks for metallic Ni and Co in the electrocatalysts containing both metals suggests that their synergistic effect may be responsible for this phenomenon. The coexistence of Ni and Co could create interactions that support crystal structure formation during synthesis.

**Figure 6:**
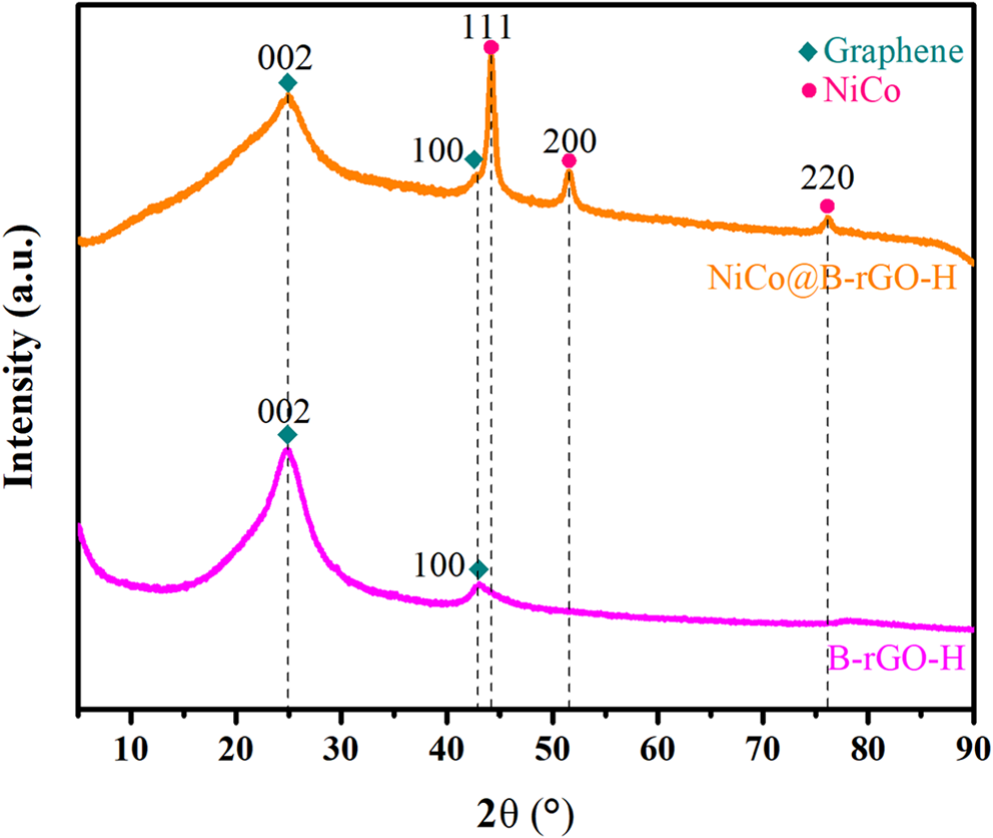
Wide-angle XRD spectra of B-rGO-H and NiCo@B-rGO-H electrocatalysts.

The SEM technique was used to determine the surface morphology and metal distributions of the GO, rGO-H, B-rGO-H, and NiCo@B-rGO-H electrocatalysts. SEM images of GO (Figure 7a–c) show a thin and layered structure. When the SEM images of rGO-H and B-rGO-H (Figure 7d–f,g–i) materials are examined, a more wrinkled and defective structure is observed compared to GO. These morphological features may result from the removal of oxygen groups through the hydrothermal process and the defects introduced by boron atom doping. Raman spectroscopy findings validate the defective structure in the morphology of rGO-H and B-rGO-H.

**Figure 7:**
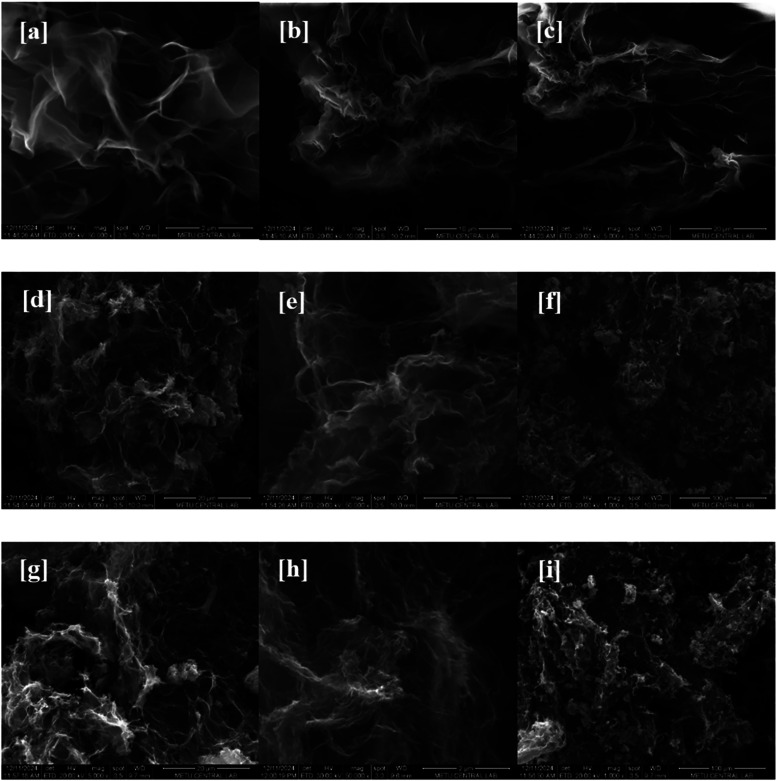
(a–c) SEM images of GO at different magnifications, (d–f) SEM images of rGO-H at different magnifications, (g–i) SEM images of B-rGO-H at different magnifications.

SEM and HR-TEM images images of the NiCo@B-rGO-H electrocatalyst are presented in Figure 8. These images reveal that metals are in nanoparticle form. Based on the SEM and HR-TEM images, the nanoparticle sizes observed in the NiCo@B-rGO-H electrocatalysts were determined to be approximately 70–100 nm (Figure 8c,f). After metal loading on B-rGO-H material using the modified polyol method, a mostly homogeneous metal distribution was observed in the SEM and HR-TEM images (Figure 8a–c,d–f). Figure 8g–m demonstrates a scanning SEM image and corresponding energy dispersive X-ray spectrum (EDX) mapping of NiCo@B-rGO-H (Figure 8g–l), revealing that Ni, Co, C, O, and N are homogeneously distributed in the structure. Additionally, it was observed that the morphology was preserved after applying the modified polyol method, indicating that metal loading did not cause any degradation.

**Figure 8:**
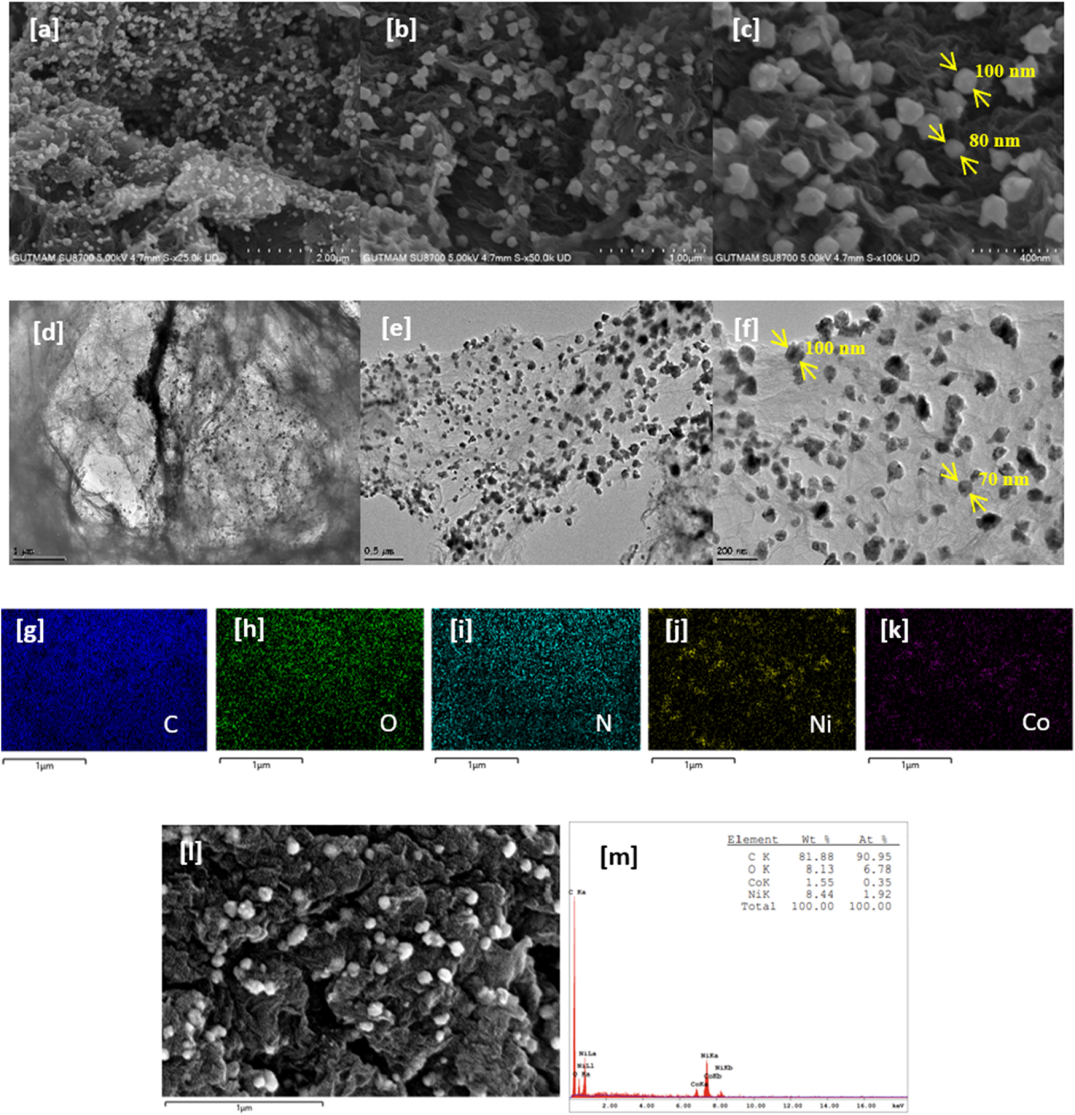
(a–c) SEM images of NiCo@B-rGO-H electrocatalyst at different magnifications, (d–f) HR-TEM images of NiCo@B-rGO-H, (l–m) EDS mapping of elemental distributions of NiCo@B-rGO-H electrocatalyst.

Figure 9 shows an HR-TEM image of the NiCo@B-rGO-H electrocatalyst, indicating that the number of layers of graphene nanosheets is 9–11. The high-resolution transmission electron microscopy (HRTEM) image further reveals the core–shell structure, with the lattice spacing of the nanoparticles measured at 0.21 nm, which corresponds to the (111) plane of the NiCo nanoparticles (Figure 9d). The shell is identified as graphene layers exhibiting an interlayer distance of approximately 0.35 nm (Figure 9).

**Figure 9:**
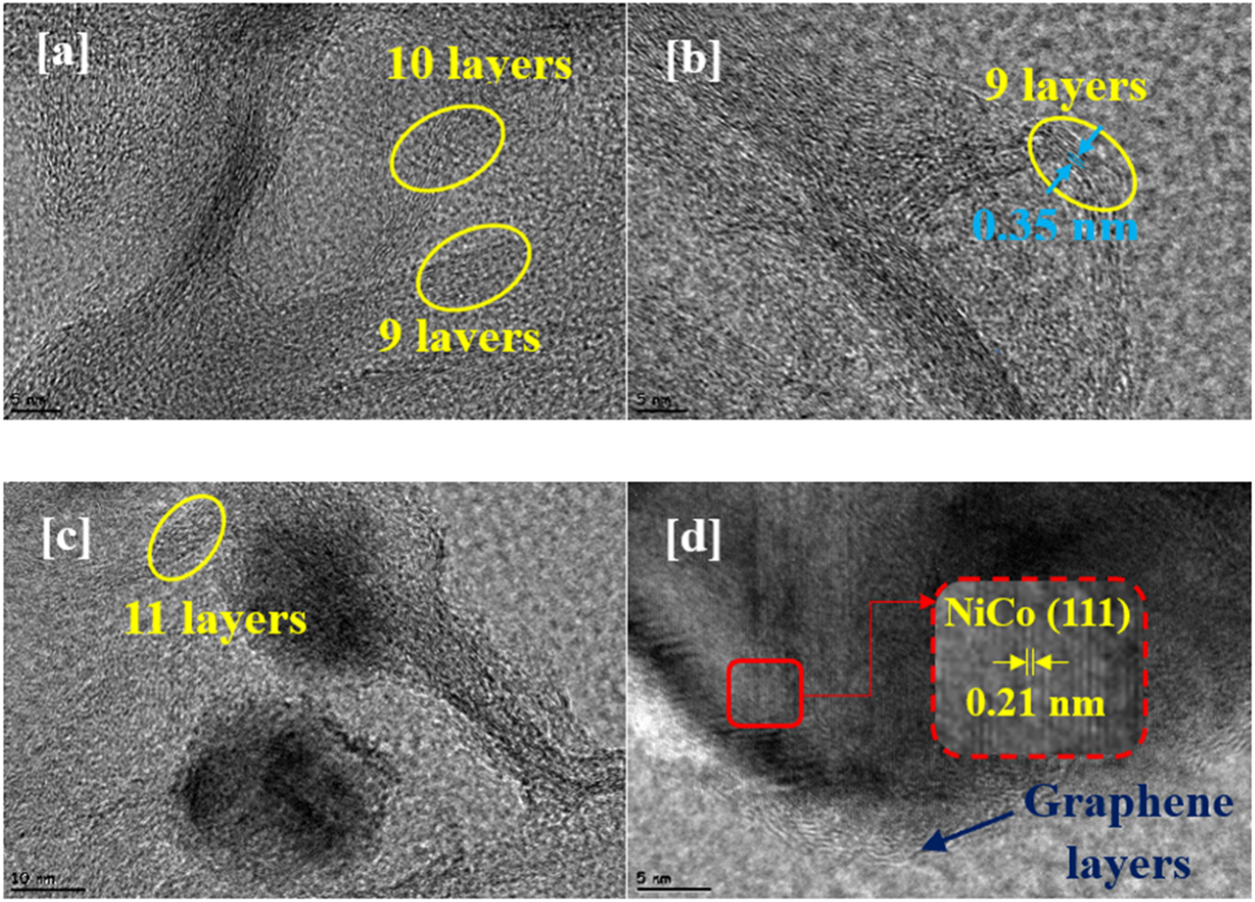
(a–d) HR-TEM images of NiCo@B-rGO-H at different magnifications.

Energy Dispersive X-ray Spectroscopy (EDS) was used alongside SEM to determine the chemical composition of the prepared materials. EDS analysis results for the prepared materials are given in Table 3. The mass compositions determined by EDS analysis for GO, rGO-H, B-rGO-H, and NiCo@B-rGO-H electrocatalysts are consistent with the mass surface compositions determined by XPS analysis.

**Table 3:** Mass Surface Compositions Determined by SEM-EDS Analysis

electrocatalyst	wt %C	wt %O	wt %Ni	wt %Co
GO	65.26	34.74		
rGO-H	86.65	13.35		
B-rGO-H	87.59	12.41		
NiCo@B-rGO-H	81.88	8.13	8.44	1.55

X-ray photoelectron spectroscopy (XPS) measurements were performed to determine the chemical composition and bonding of the atoms in NiCo@B-rGO-H. The Ni 2p XPS spectrum (Figure 10a) reveals two spin–orbit split doublets accompanied by satellite peaks. The Ni 2p_3_/_2_ region was deconvoluted into two distinct components corresponding to metallic Ni^0^ (853.0 eV) and oxidized Ni^2+^ (854.8 eV), along with a characteristic satellite peak at 870.4 eV. The metallic Ni^0^ peak observed at 853.0 eV confirms that nickel predominantly exists in the zerovalent metallic state (Ni^0^) and is successfully incorporated into boron-doped graphene (B-rGO-H).^36^ Furthermore, the peak observed at 854.8 eV can be assigned to NiO or Ni­(OH)_2_ species. In contrast, the Ni 2p_1/2_ region exhibited substantial overlap between species; therefore, it was modeled using a single broad peak to represent both states with an accompanying satellite peak at 873.5 eV. Co XPS spectra are shown in Figure 10b. The peaks at 778.3 and 780.2 eV correspond to Co 2p_3/2_ for Co^0^ and Co^2+^, respectively. The peaks at 778.3 and 793.8 eV are Co 2p_3/2_ and Co 2p_1/2_ peaks for metallic Co. The presence of a satellite peak at 784.0 eV further supports the existence of Co^2+^ species, typically attributed to CoO or Co­(OH)_2_.^37^ Due to significant peak overlap in the Co 2p_1_/_2_ region, a single broad component at 794.5 eV was used to represent both oxidation states. Although no phases other than metallic Ni and Co were observed in the XRD analysis, the presence of Ni^2+^ and Co^2+^ in the XPS spectra is thought to result from the oxidation of surface metallic Ni and Co due to the air exposure.^38,39^ The C 1s XPS spectrum (Figure 10c) was deconvoluted into six distinct components centered at 284.5, 285.0, 286.5, 288.0, 289.0, and 290.9 eV, corresponding to C=C, C–C/C=N, C–O/C–N, C=O, and O–C=O bonding, respectively.^40^ The N 1s XPS spectrum can be split into five individual peaks (Figure 10d). In the N 1s XPS spectrum, the peaks observed at approximately 398.3, 399.2, 400.1, 401.5, and 403.5 eV are attributed to pyridinic, pyrrolic, graphitic, quaternary, and oxidized nitrogen species, respectively, confirming the nitrogen incorporation within the graphene matrix.^41,42^ In the B 1s XPS spectrum (Figure 10e), the peak observed at 192.0 eV corresponds to the BC_3_ structure, confirming the substitutional doping of boron into the graphene framework.^43^ The O 1s XPS spectrum (Figure 10f) was deconvoluted into four peaks centered at 530.2, 531.6, 533.0, and 534.7 eV, corresponding to metal oxides, metal hydroxides, C=O/O–C=O, C–O, and adsorbed H_2_O, respectively.^44,45^

**Figure 10:**
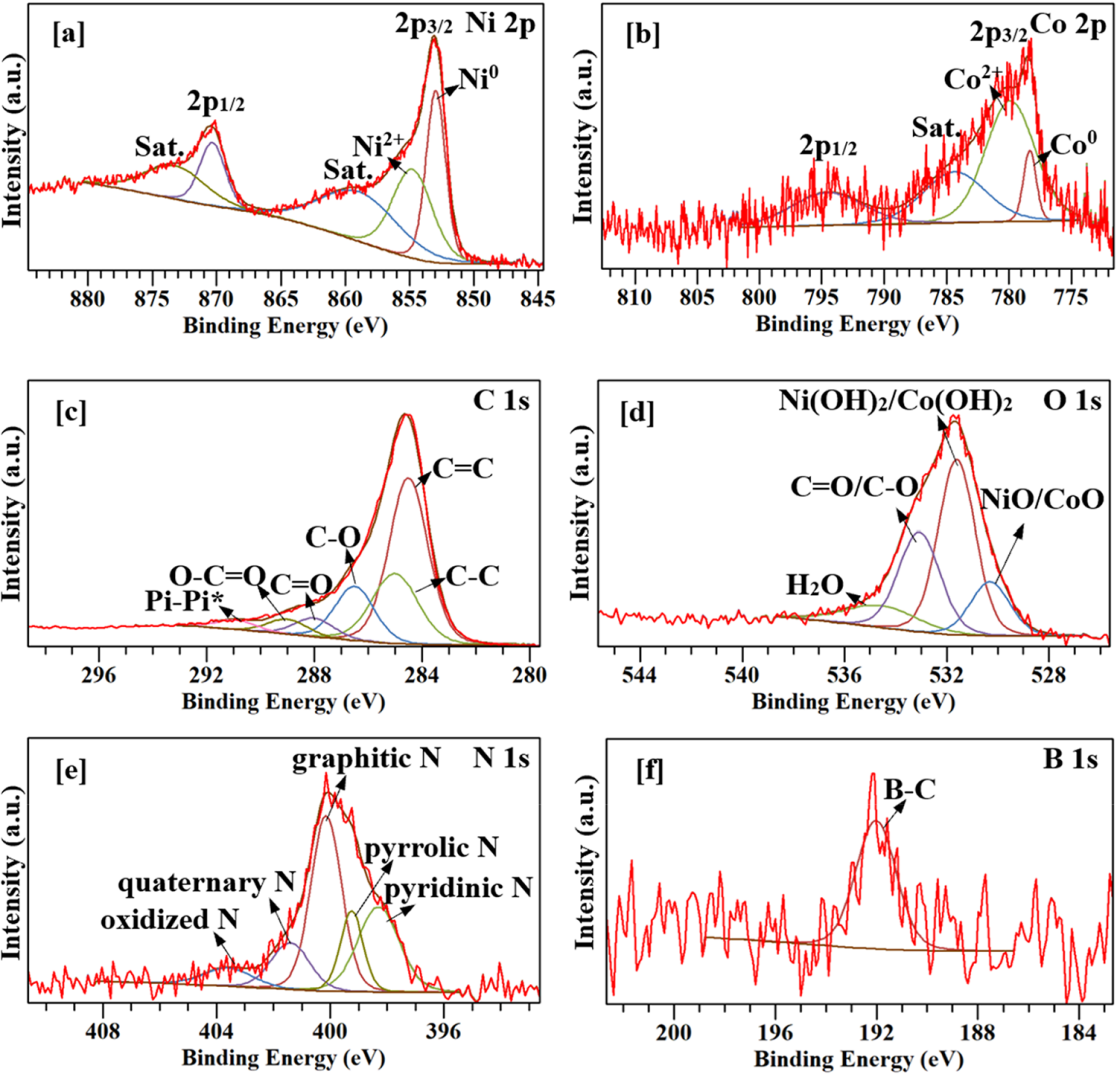
XPS spectra of NiCo@B-rGO-H; (a) Ni 2p, (b) Co 2p, (c) C 1s, (d) O 1s, (e) N 1s, (f) B 1s

To examine the surface chemical changes of the catalyst after electrochemical operation, post-HER XPS analyses were performed (Figure S1). The Ni 2p and Co 2p spectra reveal that both Ni and Co undergo partial surface oxidation to Ni­(OH)_2_/NiOOH and Co­(OH)_2_/CoOOH species during the HER process, indicating the formation of active oxyhydroxide phases commonly associated with enhanced alkaline hydrogen evolution kinetics.

### 3.2 Electrocatalytic HER Performance

Electrochemical measurements were performed in 1 M KOH solution by linear sweep voltammetry (LSV), electrochemical impedance spectroscopy (EIS), cyclic voltammetry, and chronopotentiometry techniques. The polarization curves of the synthesized electrocatalysts are given in Figure 11a. For each electrocatalyst, the potential at a current density of 10 mA cm^–2^ was read, and overpotential values were determined. The overpotential of the empty GC electrode was measured as 800 mV. The overpotential values of rGO-H and B-rGO-H, synthesized by the hydrothermal method, were read as 656 and 556 mV, respectively. It was observed that the overpotential decreased to 100 mV with graphene B doping. This improvement is attributed to improving the electrocatalytic performance by enhancing the electrical properties of the rGO. For electrocatalysts, Ni@B-rGO-H (409 mV), Co@B-rGO-H (482 mV), and NiCo@B-rGO-H (242 mV) hydrogen evolution reaction overpotential values at 10 mA cm^–2^ current density were determined and shown in Figure 11d. While Ni@B-rGO-H and Co@B-rGO-H electrocatalysts alone have overpotential values of 409 and 482 mV, respectively, for the NiCo@B-rGO-H electrocatalyst, the overpotential required for the hydrogen production reaction decreased to 242 mV. This observation shows that a synergistic effect occurs between Ni and Co in the synthesized electrocatalyst, improving the electrocatalytic performance.

**Figure 11:**
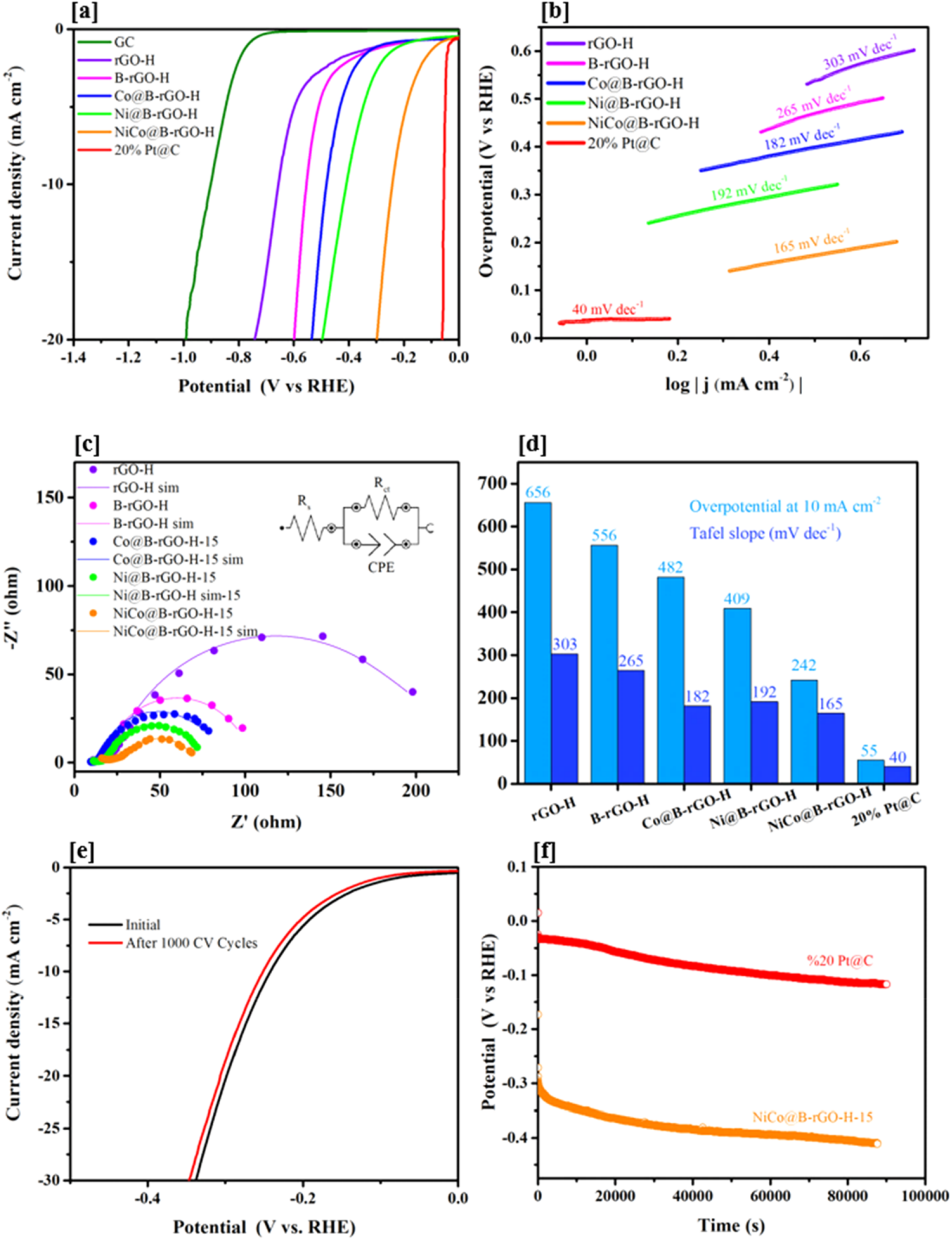
(a) Polarization curves of the synthesized electrocatalysts and 20% Pt@C (b) the corresponding Tafel plots (c) Nyquist curves of the synthesized electrocatalysts (d) the corresponding overpotentials at 10 mA cm^–2^ (e) the chronopotentiometric (*E* – *t*) curves measured at the constant current density of 10 mA cm^–2^ for 24 h (f) polarization curves of NiCo@B-rGO-H initially and after 1000 cycles.

Tafel slopes were determined by drawing Tafel curves for each electrocatalyst synthesized using the data forming the polarization curves obtained by the linear scanning voltammetry (Figure 11b). Tafel curves were obtained by plotting the overpotential against the logarithm of the current density. An ideal electrocatalyst should have small overpotential and Tafel slope values at a given current density. Tafel slope values of the electrocatalysts are in the order from largest to smallest: rGO-H > B-rGO-H > Co@B-rGO-H > Ni@B-rGO-H > NiCo@B-rGO-H. The results obtained support the overpotential values of electrocatalysts. In alkaline media, the hydrogen evolution reaction (HER) generally follows the Volmer step, succeeded by either the Heyrovsky or Tafel step. Typically, Tafel slopes of approximately 120, 40, and 30 mV dec^–1^ correspond to Volmer-, Heyrovsky-, and Tafel-limited kinetics, respectively.^46^ In this study, the measured Tafel slope of 165 mV dec^–1^ for the NiCo@B-rGO-H catalyst suggests a Volmer–Heyrovsky pathway dominated by the Volmer (water dissociation and hydrogen adsorption) step.

To further substantiate the influence of boron incorporation, comparative electrochemical and structural analyses for the boron-free NiCo@rGO-H catalyst are provided in the Figures S2–S4. As shown in Figure S2, the polarization curves demonstrate that the boron-doped NiCo@B-rGO-H catalyst exhibits a lower overpotential of 242 mV at 10 mA cm^–2^, compared with 360 mV for the undoped NiCo@rGO-H catalyst, confirming the beneficial effect of boron on HER activity. Correspondingly, the Tafel slope decreases from 196 mV dec^–1^ (NiCo@rGO-H) to 165 mV dec^–1^ (NiCo@B-rGO-H), as presented in Figure S3, indicating faster HER kinetics facilitated by boron-induced electronic modulation. The XRD patterns (Figure S3) reveal similar diffraction peaks for both samples corresponding to metallic Ni and Co phases, yet without any boron-related crystalline peaks, confirming that boron is incorporated into the carbon framework rather than forming a separate phase. Furthermore, the XPS spectra (Figure S4) show characteristic Ni 2p and Co 2p features for both electrocatalysts, while only the boron-doped sample displays a distinct B 1s peak at 192.0 eV, consistent with BC_3_-type bonding. Together, these results confirm that boron doping preserves the metallic Ni–Co active structure while introducing electronic and defect-level modifications that promote charge transfer and accelerate hydrogen evolution kinetics under alkaline conditions.

Electrochemical impedance spectroscopy (EIS) measurements were conducted within a broad frequency range of 0.01 to 10^5^ Hz to assess the interfacial electrochemical behavior of various electrocatalysts synthesized on reduced graphene oxide (rGO). Nyquist plots were obtained using electrochemical impedance spectroscopy (EIS). The Nyquist plots (Figure 11c) display depressed semicircles, indicating mixed charge transfer and capacitive behavior across the electrode–electrolyte interface. To interpret the experimental data, the Nova software was utilized to simulate the impedance spectra using the nonlinear least-squares (NLLS) fitting method. The best fit was obtained using the equivalent circuit model depicted in the inset of Figure 11c, comprising three representative circuit elements, which are *R*_s_, CPE, and *R*_ct_. *R*_s_ is the solution resistance, corresponding to the intercept of the semicircle on the *Z*′ axis. CPE is the constant phase element representing nonideal capacitive behavior due to surface roughness, porosity, and inhomogeneity, which is related to the double layer capacitance (*C*_dl_). *R*_ct_ is the charge transfer resistance, related to the electron transfer kinetics at the electrode–electrolyte interface. The *R*_s_, *R*_ct_, Yo, *n*, and *C*_dl_ values obtained from the equivalent circuit simulations for each electrocatalyst are summarized in Table 4. A decrease in *R*_ct_ and an increase in double-layer capacitance (*C*_dl_) indicate enhanced electrocatalytic performance. The *R*_ct_ value is related to the electron transfer ability of the electrocatalyst under working conditions. Notably, the charge transfer resistance varied significantly among the samples, offering insight into the electrocatalytic activity. rGO-H exhibited the highest *R*_ct_ (183.54 Ω), indicating sluggish charge transfer kinetics, consistent with its low intrinsic catalytic activity. Upon boron doping (B-rGO-H), *R*_ct_ markedly decreased to 84.11 Ω, suggesting improved conductivity and faster interfacial electron exchange. The incorporation of Ni and Co metals further reduced *R*_ct_ values significantly (Ni@B-rGO-H: 58.91 Ω, Co@B-rGO-H: 67.15 Ω, NiCo@B-rGO-H: 51.24 Ω). These results highlight the enhanced charge transfer facilitated by the conductive rGO matrix and the synergistic effects of bimetallic sites. Particularly, the NiCo@B-rGO-H catalyst exhibited the *R*_ct_, demonstrating superior HER kinetics, in agreement with its highest *C*_dl_ value (27.7 mF/cm^2^), implying a large electrochemical surface area (ECSA) and rapid ion transfer.^47,48^ It was observed that the B doping in rGO-H led to an increase in the double-layer capacitance (*C*_dl_) from 6.11 to 9.63 mF/cm^2^. The introduction of transition metals such as Ni and Co, especially in the NiCo@B-rGO-H composite, significantly enhanced the electrochemical surface area (ECSA), as reflected by the increased *C*_dl_ values. This enhancement is associated with improved electrical conductivity, better ion accessibility, and synergistic effects between rGO and metal active sites.^27,49–51^ The CPE exponent n ranged from 0.73 to 0.89, indicating a deviation from ideal capacitive behavior, commonly associated with rough or porous electrode surfaces. Examination of the EIS results for all synthesized electrocatalysts indicates good agreement with the determined overpotential and Tafel slope values, confirming their correlation with electrocatalytic activity.

**Table 4:** Calculated Values of the EIS Circuit Elements for the Equivalent Circuit of rGO-H, B-rGO-H, Ni@B-rGO-H, Co@B-rGO-H, and NiCo@B-rGO-H in 1 M KOH Solution for HER

electrocatalyst	*R*_s_ (Ω)	*R*_ct_ (Ω)	*Y*_o_ (S·s*^n^*)	*n*	*C*_dl_ (mF)	*C*_dl_ (mF/cm^2^)
rGO-H	11.46	183.54	2.98 × 10^–3^	0.842	0.43	6.11
B-rGO-H	11.30	84.11	2.72 × 10^–3^	0.887	0.68	9.63
Ni@B-rGO-H	11.41	58.91	4.34 × 10^–3^	0.782	1.71	24.10
Co@B-rGO-H	9.85	67.15	3.01 × 10^–3^	0.779	1.50	21.14
NiCo@B-rGO-H	16.25	51.24	6.33 × 10^–3^	0.733	1.97	27.70

The electrochemical stability of the synthesized NiCo@B-rGO-H electrocatalyst was systematically evaluated through CV and chronoamperometry tests. As shown in Figure 11e, the LSV polarization curves of NiCo@B-rGO-H before and after 1000 continuous CV cycles at a scan rate of 50 mV s^–1^ in 1 M KOH display a negligible change in HER activity. The overpotential at a current density of 10 mA cm^–2^ shows only a slight increase (∼10 mV), confirming the excellent electrochemical stability of the catalyst under prolonged redox cycling. This indicates that the hybrid structure of boron-doped reduced graphene oxide decorated with NiCo nanoparticles maintains its active sites and structural integrity throughout the stability test period. To further evaluate the long-term electrochemical stability of the NiCo@B-rGO-H electrocatalyst, a chronopotentiometry (CP) test was performed at a constant current density of 10 mA cm^–2^ for 24 h. The resulting potential–time profile is presented in Figure 11f. During the 24-h operation period, a gradual yet noticeable decline in potential was observed. Initially recorded around −0.30 V, the potential gradually decreased to approximately −0.40 V over time. This shift may be the accumulation of gas bubbles at the electrode–electrolyte interface that hinders catalytic efficiency, considering the fact that a comparable potential drop was also observed for the commercial 20 wt % Pt@C electrocatalyst. The results clearly indicate that the NiCo@B-rGO-H electrocatalyst is capable of maintaining its operational stability for long-term hydrogen evolution. Despite the gradual potential decay, the similarity in behavior with the benchmark 20% Pt@C electrocatalyst further highlights the high stability of the synthesized material under continuous HER conditions.

Despite the higher overpotential compared to 20%Pt@C, the NiCo@B-rGO-H electrocatalyst exhibits remarkable stability and durability, making it a promising noble-metal-free alternative for alkaline hydrogen evolution applications. The synergistic interaction between Ni and Co nanoparticles and the boron-doped rGO matrix contributes to the electrochemical stability of the electrocatalyst. A comparison table summarizing the overpotential and Tafel slope values of various reported Ni–Co/carbon-based catalysts has been prepared and added to the **Supporting Information** as Table S1, where our catalyst (NiCo@B-rGO-H) is listed alongside previously reported systems for direct comparison.

## 4 Conclusion

In this study, Ni@B-rGO-H, Co@B-rGO-H, and NiCo@B-rGO-H electrocatalysts were synthesized using the modified polyol method for use in hydrogen production via alkaline water electrolysis. In conclusion, NiCo@B-rGO-H electrocatalyst exhibits the highest activity for HER, evidenced by the lower overpotential value and Tafel slope (242 mV, 165 mV/dec). The results demonstrate that the NiCo@B-rGO-H electrocatalyst exhibits promising electrocatalytic activity and high stability for HER in alkaline water. Boron doping improves the electrocatalytic performance by tuning the electronic properties of graphene layers. Increasing the total metal content in the electrocatalyst has improved the electrochemical performance as expected. The synergistic effect between Ni and Co in the synthesized electrocatalyst improves the electrocatalytic performance. Overall, the characterization and electrochemical measurement studies reveal that the used procedure is a successful way to prepare efficient and stable heteroatom-doped graphene-supported nonprecious metal nanoparticle electrocatalyst for alkaline water electrolysis and various catalytic applications.

In future studies, the optimization of metal loading ratios and particle size distributions could be systematically investigated to establish quantitative correlations between composition, morphology, and catalytic activity. Additionally, the influence of boron doping level and different heteroatom codopants (e.g., N or P) on the electronic structure of graphene could be explored to further enhance the intrinsic activity of the Ni–Co sites. Extending the application of the NiCo@B-rGO-H electrocatalyst to overall water splitting and integrating it into practical alkaline electrolyzer systems would also be valuable for evaluating its long-term durability and scalability.

## Supplementary Material



## Data Availability

All experimental data and characterization results supporting the findings of this work are included within the article and the Supporting Information files.

## Notes

Additional SEM, XPS, and electrochemical characterization data (Figures S1–S5) supporting the findings of this study (PDF)
