# Supplementary material for: Synergistic Effect of Nickel and Cobalt Nanoparticles Anchored on Boron-Doped Reduced Graphene Oxide for Enhanced Alkaline Hydrogen Evolution Reaction
Source: ACS Omega. 2025 Nov 20;10(48):59314–27. doi: 10.1021/acsomega.5c08749 (PMC12771462; doi:10.1021/acsomega.5c08749)
Supplement: Supplementary file 1 [file ao5c08749_si_001.pdf]

Supporting Information

Synergistic Effect of Nickel and Cobalt Nanoparticles Anchored on Boron-Doped Reduced Graphene Oxide for Enhanced Alkaline Hydrogen Evolution Reaction

Nida Arasan[a*] and Fatih Akkurt[a]

[a] Department of Chemical Engineering, Faculty of Engineering,

Gazi University, Ankara 06570, Turkey

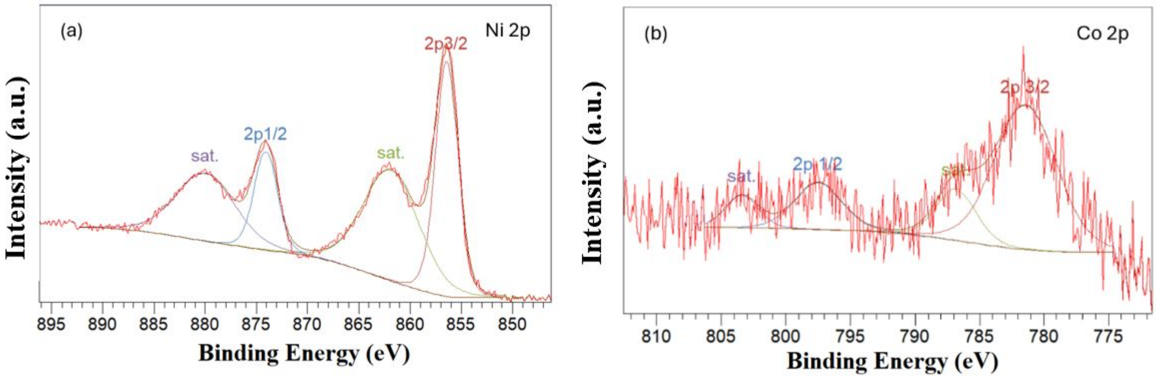

Figure S1. Post-reaction XPS spectra of NiCo@B-rGO-H ; (a) Ni 2p, (b) Co 2p

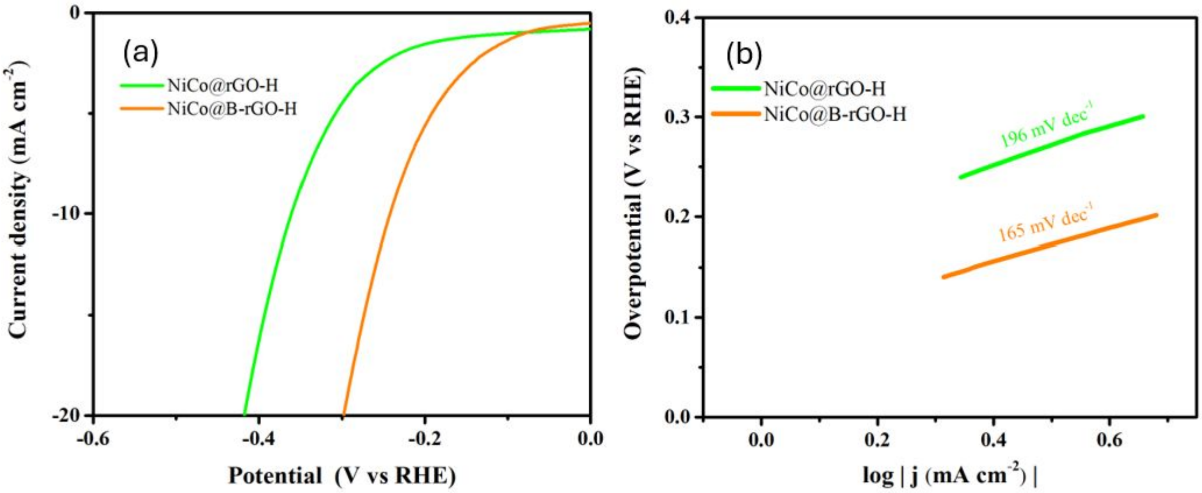

Figure S2. (a) Polarization curves of the NiCo@rGO-H, and NiCo@B-rGO-H electrocatalysts, (b) Tafel plots of the NiCo@rGO-H, and NiCo@B-rGO-H electrocatalysts.

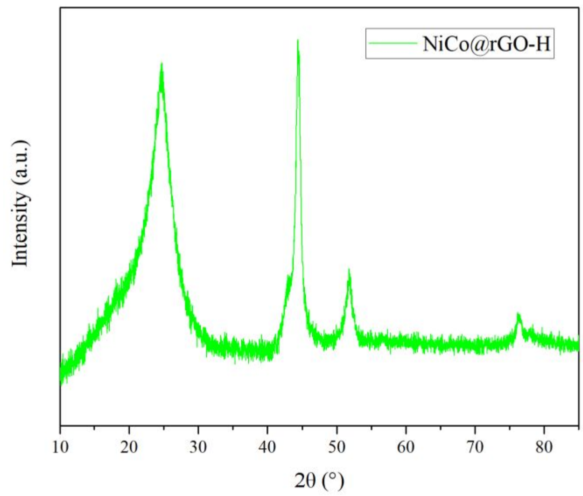

Figure S3. XRD pattern of NiCo@rGO-H electrocatalyst.

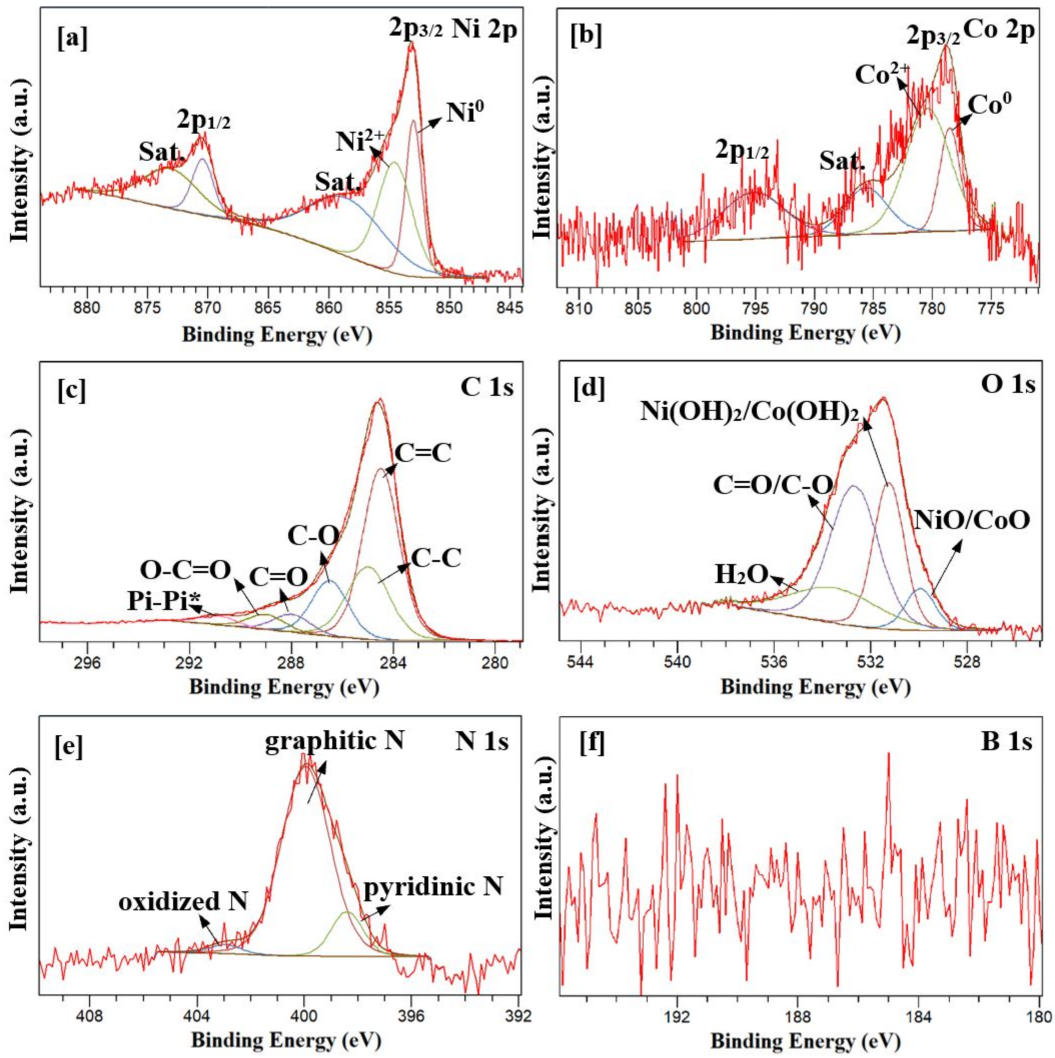

Figure S4. XPS spectra of NiCo@rGO-H; (a) Ni 2p, (b) Co 2p, (c) C 1s, (d) O 1s, (e) N 1s, (f) B 1s

Table S1. Summary of HER overpotential and Tafel slope values for reported Ni-Co/Carbon-based catalysts in 1 M KOH

**Catalyst**	**Electrolyte**	**Overpotential @10 mA cm⁻² (mV)**	**Tafel Slope (mV dec⁻¹)**	**Reference**
NiCo@B-rGO-H	1 M KOH	242	165	This work
$MNi_{63}Co_{37}/rGO_5$	1 M KOH	115	45	1
Ni/Co/1:4-coated graphene	1 M KOH	101.92	164.65	2
CC@NC/NiCo-P	1 M KOH	140	79.6	3
NiCo/C	1 M KOH	21	14	4
NiCo/rGO	1 M KOH	194	114.8	5
C@NiCo11	1 M KOH	232	157	6
NiCo-NCP/CNT	1 M KOH	63	54	7
NiCo-N-CNTs	1 M KOH	54	41.1	8
NiCo-SAD-NC	1 M KOH	61	55	9
NiCoP HA/CC	1 M KOH	89	99.8	10

References

1. Dong, J.; Sun, T.; Zhang, Y.; Zhang, H.; Lu, S.; Hu, D.; & Xu, L. Mesoporous NiCo alloy/reduced graphene oxide nanocomposites as efficient hydrogen evolution catalysts. *J. Colloid Interface Sci. 2021*, *599*, 603-610. https://doi.org/10.1016/j.jcis.2021.04.124

2. Huner, B.; Demir, N.; & Kaya, M. F. Hydrogen evolution reaction performance of Ni–Co-coated graphene-based 3D printed electrodes. *ACS omega 2023*, *8*(6), 5958-5974. https://doi.org/10.1021/acsomega.2c07856

3. Zhang, Z.; Song, N.; Wang, J.; Liu, Y.; Dai, Z.; & Nie, G. Polydopamine-derived carbon layer anchoring NiCo-P nanowire arrays for high-performance binder-free supercapacitor and electrocatalytic hydrogen evolution. *SusMat 2022*, *2*(5), 646-657. https://doi.org/10.1002/sus2.49

4. Jia, J.; Wang, R.; She, C.; Li, C.; Niu, Y.; Hu, B.; & Dong, H. Carbon supported NiCo alloy catalyst with face-to-face encapsulated structure for electrocatalytic hydrogen evolution. *Int. J. Hydrogen Energy 2025*, *131*, 20-25. https://doi.org/10.1016/j.ijhydene.2025.04.296

5. Kamali, S.; Zhiani, M.; & Tavakol, H. Synergism effect of first row transition metals in experimental and theoretical activity of NiM/rGO alloys at hydrogen evolution reaction in alkaline electrolyzer. *Renew energy 2020*, *154*, 1122-1131. https://doi.org/10.1016/j.renene.2020.03.031

6. Tan, S.; Ouyang, W.; Ji, Y.; Hong, Q. Carbon Wrapped Bimetallic NiCo Nanospheres Toward Excellent HER and OER Performance. J. Alloys Compd. 2021, 889, No. 161528. https://doi.org/10.1016/j.jallcom.2021.161528

7. Sun, M.; Yun, S.; Dang, J.; Zhang, Y.; Liu, Z.; & Qiao, D. 1D/3D rambutan-like Mott–Schottky porous carbon polyhedrons for efficient tri-iodide reduction and hydrogen evolution reaction. *Chem. Eng. J. 2023*, *458*, 141301. https://doi.org/10.1016/j.cej.2023.141301

8. Balasubramanian, P.; Khan, H.; Baek, J. H.; & Kwon, S. H. Binary NiCo nanoalloys and single-atoms implanted nitrogen-doped carbon nanotubes as highly efficient, robust electrocatalyst for overall water splitting. *Chem. Eng. J. 2023*, *471*, 144378. https://doi.org/10.1016/j.cej.2023.144378

9. Kumar, A.; Bui, V. Q.; Lee, J.; Wang, L.; Jadhav, A. R.; Liu, X.; & Lee, H. Moving beyond bimetallic-alloy to single-atom dimer atomic-interface for all-pH hydrogen evolution. *Nat. Commun. 2021*, *12*(1), 6766. https://doi.org/10.1038/s41467-021-27145-3

10. Ma, S.; Wang, L.; Zhang, S.; Jin, H.; Wan, M.; Pan, Y.; & Du, M. Facile fabrication of a binary NiCo phosphide with hierarchical architecture for efficient hydrogen evolution reactions. *Int. J. Hydrogen Energy 2019*, *44*(8), 4188-4196. https://doi.org/10.1016/j.ijhydene.2018.12.133
